# The influence of hypoxia and energy depletion on the response of endothelial cells to the vascular disrupting agent combretastatin A-4-phosphate

**DOI:** 10.1038/s41598-020-66568-8

**Published:** 2020-06-18

**Authors:** Toby Holmes, Andrew W. Brown, Marie Suggitt, Lucy A. Shaw, Lucy Simpson, Joseph P. A. Harrity, Gillian M. Tozer, Chryso Kanthou

**Affiliations:** 1 Tumour Microcirculation Group, Department of Oncology and Metabolism, University of Sheffield, School of Medicine, Beech Hill Road, Sheffield, S10 2RX United Kingdom; 2 Department of Chemistry, University of Sheffield, Dainton Building, Brook Hill, Sheffield, S3 7HF United Kingdom

**Keywords:** Cancer microenvironment, Targeted therapies, Tumour angiogenesis, Actin, Microtubules

## Abstract

Combretastatin A-4 phosphate (CA4P) is a microtubule-disrupting tumour-selective vascular disrupting agent (VDA). CA4P activates the actin-regulating RhoA-GTPase/ ROCK pathway, which is required for full vascular disruption. While hypoxia renders tumours resistant to many conventional therapies, little is known about its influence on VDA activity. Here, we found that active RhoA and ROCK effector phospho-myosin light chain (pMLC) were downregulated in endothelial cells by severe hypoxia. CA4P failed to activate RhoA/ROCK/pMLC but its activity was restored upon reoxygenation. Hypoxia also inhibited CA4P-mediated actinomyosin contractility, VE-cadherin junction disruption and permeability rise. Glucose withdrawal downregulated pMLC, and coupled with hypoxia, reduced pMLC faster and more profoundly than hypoxia alone. Concurrent inhibition of glycolysis (2-deoxy-D-glucose, 2DG) and mitochondrial respiration (rotenone) caused profound actin filament loss, blocked RhoA/ROCK signalling and rendered microtubules  CA4P-resistant. Withdrawal of the metabolism inhibitors restored the cytoskeleton and CA4P activity. The AMP-activated kinase AMPK was investigated as a potential mediator of pMLC downregulation. Pharmacological AMPK activators that generate AMP, unlike allosteric activators, downregulated pMLC but only when combined with 2DG and/or rotenone. Altogether, our results suggest that Rho/ROCK and actinomyosin contractility are regulated by AMP/ATP levels independently of AMPK, and point to hypoxia/energy depletion as potential modifiers of CA4P response.

## Introduction

Vascular disrupting agents (VDAs) cause tumour blood vessels to collapse and this strategy kills tumour cells indirectly by rapidly cutting off their blood supply^1^. CA4P (combretastatin-A4 disodium phosphate), is a member of the tubulin-binding family of VDAs, and has been tested extensively in preclinical tumour models^2–4^ and also in the clinic^5,6^. However, CA4P and other tubulin-binding VDAs have not yet gained FDA approval for the treatment of cancer despite resulting in some clinical responses when combined with additional treatments of chemotherapy or anti-angiogenic agents^7^.

Even though this area has been researched extensively for almost three decades, the molecular mechanisms through which CA4P and other tubulin-binding VDAs cause tumour blood vessels to collapse are still not fully understood^8,9^. The extent to which tumours respond to this type of therapy is also extremely variable and although progress has been made in establishing the factors that govern tumour susceptibility to VDAs^10,11^ there are still substantial gaps in our knowledge. At the cellular level, the binding of CA4P to β-tubulin disrupts endothelial microtubules, and this process rapidly activates molecular signalling pathways that modify the actin cytoskeleton and the morphology of endothelial cells, thereby severely compromising their barrier function^8,12^. RhoA-GTPase and its target kinase, Rho kinase (ROCK), are members of a major signalling pathway activated by CA4P in endothelial cells after microtubules are disrupted^8^. CA4P-activates RhoA/ROCK-mediated actinomyosin contractility and actin reorganisation through phosphorylating target protein myosin light chain (MLC), causing stress fibres to form. In some cells ROCK-dependent contractility drives a characteristic “membrane blebbing” morphology. In blebbing cells, focal adhesions are disrupted, highly contractile stress fibres misassemble into a spherical network surrounding the cytoplasm and numerous actin-rich surface blebs develop^8^. CA4P also disrupts endothelial cell-to-cell VE-cadherin junctions^13^ and within minutes the permeability of monolayers rises through processes that are Rho/ROCK dependent^8^. The effects of CA4P on endothelial morphology and permeability are postulated to contribute to tumour vascular collapse, which occurs within minutes after tumours are treated with the drug^2,14^. More recently, we showed that CA4P activates ROCK *in vivo* and ROCK is required for full tumour vascular disrupting activity^9^ thus providing the first evidence that signalling pathways identified *in vitro* relate to the drug’s rapid *in vivo* mechanism of action.

Most solid tumours contain regions of hypoxia of variable severity^15,16^. Tumours become hypoxic because the demands for oxygen placed by the rapidly proliferating cancer cells cannot be met by angiogenesis and the resulting abnormal tumour blood supply^17^. Poorly perfused regions in a tumour may also be low in nutrients such as glucose, exacerbated by high glucose uptake and consumption rates^18^. Tumour cells are well adapted to survive under low oxygen conditions^19^, and despite retaining functional mitochondria, they favour glycolysis for generating ATP by converting glucose to pyruvate and lactate, even if sufficient oxygen is present, a phenomenon known as the ‘Warburg effect’^20^. Rather surprisingly, endothelial cells from normal as well as pathological tissues also use glycolysis as a means of generating ATP and are less dependent on oxidative phosphorylation for their energy supplies^21^. Both hypoxia and energy depletion are sensed by the master switch molecule adenosine monophosphate protein kinase (AMPK). AMPK is a serine/threonine enzyme that becomes phosphorylated and activated when oxygen levels are low or when ATP levels drop and the ratio of AMP/ATP rises^22^. AMPK has many functions including a key role in regulating metabolism. Under low energy conditions it functions mainly to conserve energy and promote ATP production through decreasing anabolic processes such as protein and lipid biosynthesis and by increasing glucose uptake. AMPK also has functions that do not directly relate to metabolism and has been implicated in the regulation of pathways associated with the remodelling of the cytoskeleton^23,24^.

While severe hypoxia makes cells resistant to radiotherapy and a number of conventional chemotherapy drugs^25^, it is not known whether tumour response to tubulin binding VDAs is influenced by hypoxia. Because VDAs are more effective at eradicating the central regions of tumours that tend to be more hypoxic, while the well oxygenated tumour periphery is generally resistant^26^, there is a general assumption that these drugs work better under hypoxia. However, supporting experimental evidence for this is lacking. Tumours become even more hypoxic and nutrient depleted following VDA-induced vascular shutdown, which is a potential drawback to this type of treatment if followed by conventional therapy or if hypoxic but surviving cells become more aggressive via hypoxia-driven gene expression^10,26,27^.

In this study we analyse the signalling activity of CA4P in conditions of hypoxia and energy depletion in endothelial cells in culture. We found that prolonged and severe hypoxia is a regulator of CA4P signalling, cytoskeletal remodelling and permeability rise. The effects of hypoxia were nevertheless reversible and normal endothelial responses to CA4P could be restored rapidly following re-oxygenation. The cytoskeletal and signalling effects of hypoxia were mimicked by glucose depletion or by reducing ATP levels in the cells with inhibitors of glycolysis and oxidative phosphorylation. Furthermore, we show that although AMPK is strongly activated by hypoxia, glucose deprivation and inhibitors of endothelial metabolism, its activation *per se* is not sufficient to regulate CA4P signalling.

## Results

### Prolonged hypoxia inhibits RhoA/ROCK signalling by CA4P but re-oxygenation restores it

**Figure 1:**
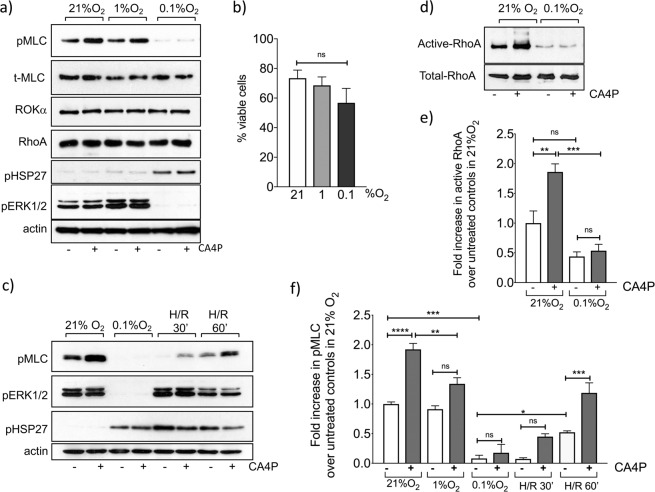
Hypoxia and hypoxia followed by re-oxygenation alter RhoA-ROCK and MAPK signalling and response to CA4P in endothelial cells. (**a**) Confluent endothelial cells were exposed to varying oxygen levels for 14 h and then CA4P (1 μM) or vehicle (PBS) were added for a further 15 min. Cells were analysed for the expression and/or phosphorylation of the indicated proteins. (**b**) Confluent cells were exposed to either 21%, 1% or 0.1% O_2_ for 14 h and then total and viable cells were counted. (**c**) Cells were exposed to 21% or 0.1% O_2_ as in (**a**) and then either immediately treated with CA4P (1 μM) or vehicle for 15 min or returned to a normal oxygen incubator for 30 or 60 min to re-oxygenate (hypoxia-re-oxygenation, H/R) before they were treated with CA4P. (**d**) Cells maintained either in 21% or 0.1% O_2_ for 14 h, were exposed to CA4P as in (**a**) and active GTP-bound RhoA was analysed in pull-down assay. (**e**) Active Rho was normalised to total RhoA and expressed as fold increase over control cells in 21% O_2_. (**f**) pMLC data from (**a**) and (**c**) was normalised to actin and expressed as fold increase over control untreated cells maintained in 21% O_2_. All data were analysed by one-way Anova followed by a Tukey post-test. Values are means ± SEM (*p < 0.05, **p < 0.01, ***p < 0.001, ****p < 0.0001). All blots and analyses are representative of 3–7 independent experiments.

Endothelial cells were exposed to varying levels of oxygen in individually gassed humidified chambers maintained within the anaerobic chamber of a hypoxia station. Control cells were maintained in a parallel chamber in 21% O_2_ to ensure that effects of gas flow and humidity were controlled accurately. Cells were treated with CA4P within the main anaerobic chamber and then returned to their corresponding individually gassed boxes for a further 15 min. The activity of CA4P was initially measured by analysing dually phosphorylated myosin light chain (pMLC), a target of ROCK^8^. Hypoxia (0.1%, 1% or 5% O_2_) for 1–5 hours had no significant effect on either basal or CA4P-induced levels of pMLC (data not shown). However, under prolonged hypoxia (1% O_2_ for 14 h), basal levels of pMLC remained the same as in normoxia but the induction of pMLC by CA4P was significantly attenuated (Fig. 1a,f). Under extreme hypoxic conditions (0.1% O_2_ for 14 h), basal pMLC was reduced to very low levels and CA4P did not induce it further (Fig. 1a,f). Levels of total MLC protein were unaffected and similarly, RhoA and ROCK isoform ROKa (ROCK2) levels were not significantly changed by hypoxia (Fig. 1a), so transcriptional regulation of these key proteins of the pathway was unlikely to be the cause of the observed reduction in MLC phosphorylation. In parallel with a reduction in MLC phosphorylation, the phosphorylation of extracellular regulated kinases (pERK1/2) was induced in 1% O_2_ while their levels dropped significantly in 0.1% O_2_ (Fig. 1a). In parallel, levels of phosphorylated heat shock protein-27 (pHSP27), a stress-induced protein and regulator of actin remodelling^28^ rose in hypoxia (Fig. 1a). Cell viability tended to decrease after prolonged severe hypoxia, although not significantly (Fig. 1b). These results agree with previous reports showing that endothelial cells are relatively resistant to hypoxic-induced cell death^29,30^. Therefore, the observed reduction in the ability of cells to respond to CA4P could not be attributed to cell death. Indeed, cells regained their responsiveness to CA4P upon re-oxygenation following severe hypoxia (Fig. 1c,f). Levels of pERK1/2 were also restored although pHP27 remained high until at least 60 min after re-oxygenation.

Upstream of ROCK, levels of active RhoA, measured by pull down assays were reduced under severe hypoxia (Fig. 1d,e). CA4P induced the activation of RhoA in 21% O_2_ as expected^31^ but failed to do so under severe hypoxic conditions, thus demonstrating that the loss of active ROCK signalling was due to inactive/inactivatable RhoA, upstream of the pathway.

### Hypoxia attenuates CA4P-induced monolayer disruption, actin remodelling and rise of endothelial permeability

**Figure 2:**
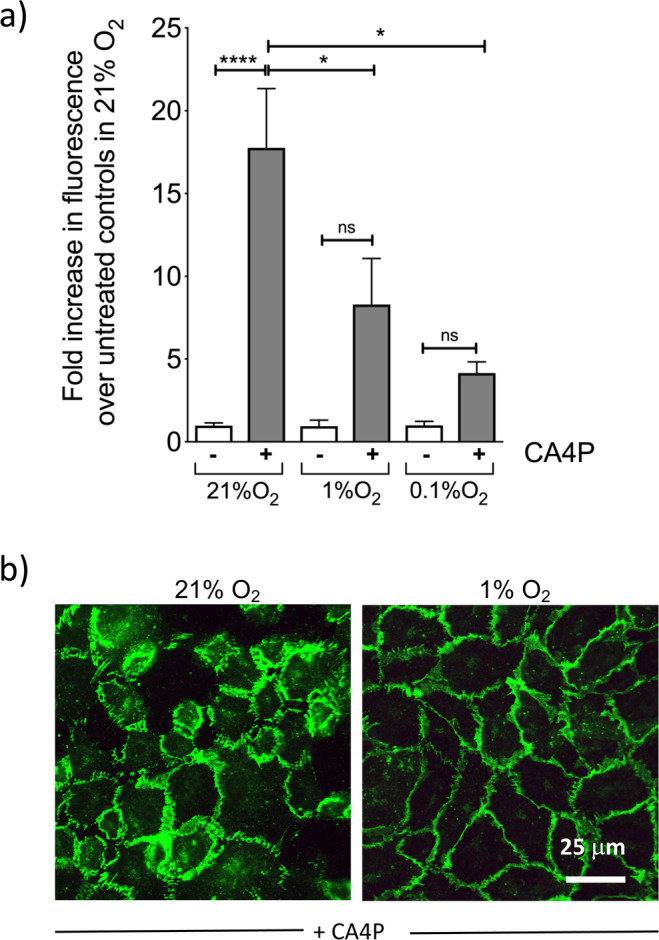
Hypoxia prevents loss of endothelial barrier function and VE-cadherin disruption mediated by CA4P. (**a**) Endothelial cells cultured to confluence in 3 μm pore size filter inserts set in companion 24-well plates were exposed to the indicated oxygen levels for 14 h and then to CA4P (1 μM) for an further 15 min. FITC dextran diffusion through the monolayers during 30 min, was expressed as fold change over control untreated cells maintained in 21% O_2._. Results are from 5–7 independent experiments. Data were analysed by one-way Anova followed by a Tukey post-test. Values are means ± SEM (*p < 0.05, **p < 0.01, ***p < 0.001, ****p < 0.0001). (**b**) Cells in 21% or 0.1% O_2_ for 14 h were treated with CA4P as in (**a**) and then fixed and stained for VE-cadherin.

**Figure 3:**
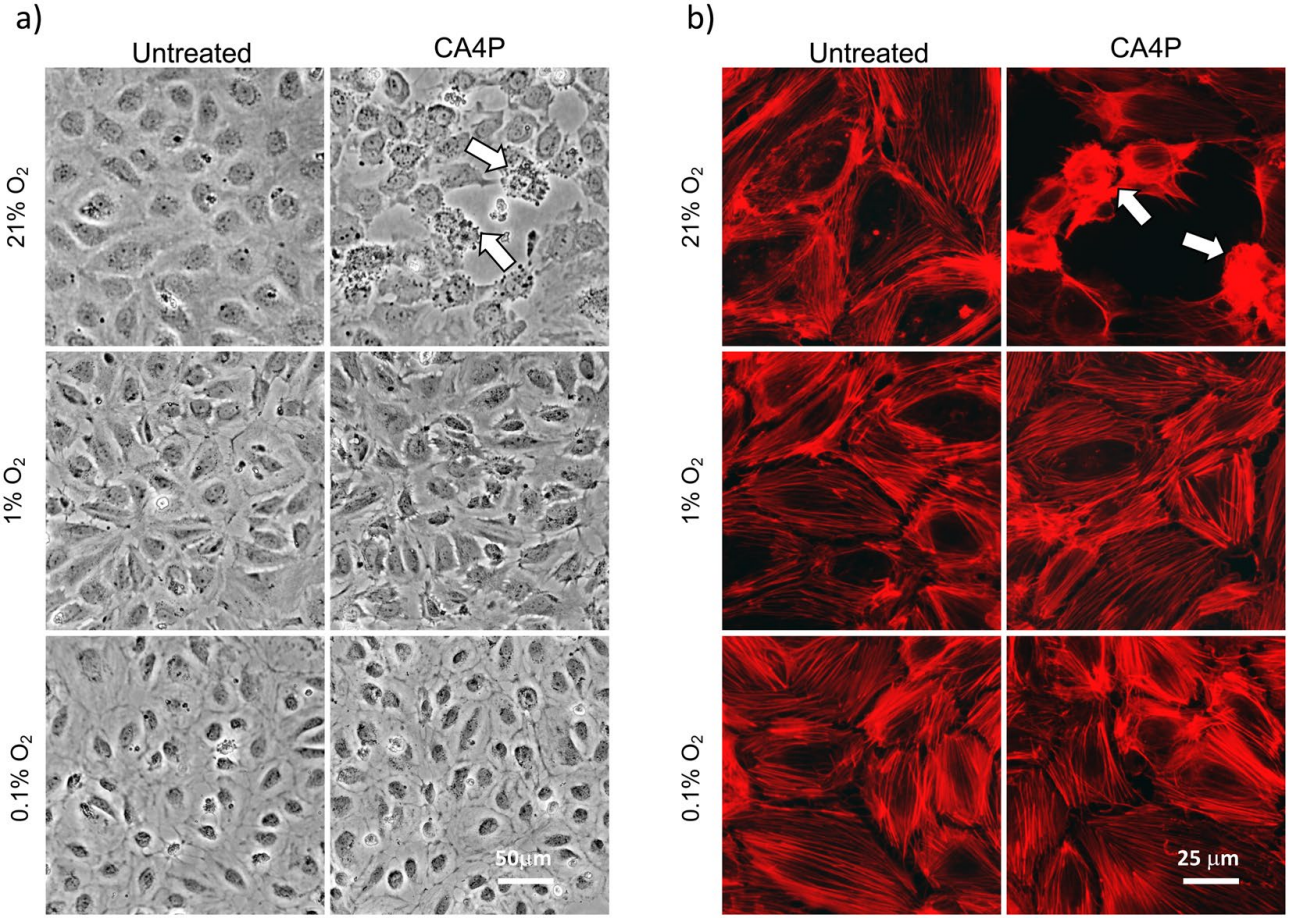
Hypoxia prevents the disruption of endothelial monolayer integrity and actin filament remodelling mediated by CA4P. (**a**) Phase contrast images of cells maintained either in 21%, 1% or 0.1% O_2_ for 14 h, and exposed to CA4P for 15 min. (**b**) cells were treated as in (**a**) and then fixed and stained with Texas-red phalloidin. Blebbing cells are indicated with white arrows in (**a**,**b**).

As shown previously, CA4P causes a rapid rise in endothelial monolayer permeability through Rho-ROCK dependent mechanisms^8^. However, endothelial monolayers were significantly less permeable to CA4P in hypoxia than in normoxia (Fig. 2a). In parallel, VE-cadherin cell-cell junctions responsible for maintaining barrier function, were also significantly less disrupted under hypoxia (Fig. 2b). Endothelial cells retract after CA4P and become contractile^8^. In hypoxia, monolayers remained intact although the cell cytoplasm appeared thin and translucent (Fig. 3a). Highly contractile ‘*blebbing*’ cells that develop soon after exposure to CA4P^8^ did not form under hypoxia (Fig. 3a,b) and this correlated with reduced levels of pMLC (see Fig. 1a) and reduced permeability. Despite complete loss of pMLC, actin stress fibers persisted under severe hypoxia (Fig. 3b), potentially driven by the high activity of pHSP27 (see Fig. 1a) which is known to cause stress fibre formation also in hypoxia^32^.

### Glucose deprivation mimics the effects of hypoxia and synergises with hypoxia to inhibit CA4P signalling

**Figure 4:**
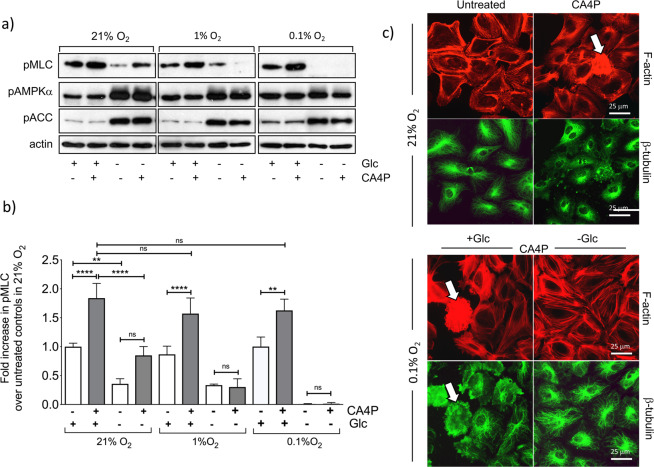
Hypoxia and glucose deprivation synergise to inhibit ROCK signalling in endothelial cells. Confluent cultures were switched to normal DMEM or DMEM without glucose (Glc), supplemented with 5% dialysed FCS and then immediately exposed to 21%, 1% or 0.1% O_2_. After 5 h, cells were treated with CA4P (1 μM) for a further 15 min. (**a**) Cell extracts were analysed for the indicated proteins. (**b**) pMLC was normalised to actin and expressed as fold increase over control cells maintained in 21% O_2_. (**c**) Cells were stained for F-actin with Texas Red phalloidin (red) and β−tubulin (green). Blebbing cells are indicated with white arrows. Results are from 3 independent experiments. Data were analysed by one-way Anova followed by a Tukey post-test. Values are means ± SEM (*p < 0.05, **p < 0.01, ***p < 0.001, ****p < 0.0001).

To investigate the effects of energy depletion on the response to CA4P, confluent cultures were transferred to glucose-free DMEM supplemented with dialysed FCS. Control cultures were maintained in similar medium but containing glucose. Cells were exposed to hypoxia or 21% O_2_ and 5 hours later were treated with CA4P. Hypoxia alone at this time point had no detectable effect on pMLC or its induction by CA4P (Fig. 4a,b). Glucose deprivation reduced both basal and CA4P-induced levels of pMLC. Under hypoxia, the effects of glucose deprivation were significantly enhanced as basal levels of pMLC declined further, and CA4P failed to induce pMLC. Glucose deprivation, and to a lesser extent hypoxia, caused the phosphorylation of AMPK and its target pACC, suggesting that the ratio of AMP/ATP increased in the cells (Fig. 4a). Contractile blebbing cells did not form in glucose-depleted media in hypoxia (0.1% O_2_) while blebbing cells were present in cultures where glucose was present (Fig. 4c). Interestingly, microtubules also appeared more resistant to CA4P-mediated disruption in cells deprived of glucose in hypoxia.

### Glycolysis and mitochondrial respiration inhibitors synergise to abolish CA4P-mediated microtubule disruption and signalling

**Figure 5:**
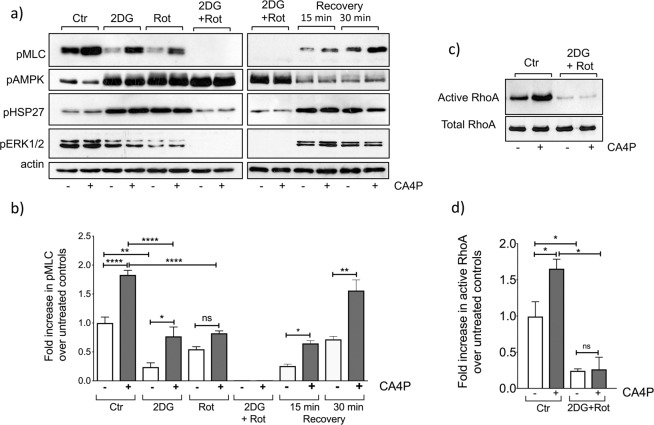
Inhibitors of glycolysis and oxidative phosphorylation inhibit pMLC signalling. (**a**) Confluent endothelial cultures were incubated with vehicle control (Ctr), 2DG (20 mM) or 0.5 μM rotenone (Rot) or 2DG plus rotenone for 1 hour. Cells were treated with CA4P (1 μM, 15 min) either immediately or after the cells were incubated in fresh media for 15 or 30 min. Cell extracts were analysed for the indicated proteins. (**b**) pMLC was normalised to actin and expressed as fold increase over untreated cells maintained in control media. (**c**) Cells were treated with 2DG and rotenone as in (**a**) and GTP-bound RhoA was analysed in pull-down assay. (**d**) Active Rho was normalised to total RhoA and expressed as fold increase over control cells in 21% O_2_. Results are from 3–7 independent experiments. Data were analysed by one-way Anova followed by a Tukey post-test. Values are means ± SEM (*p < 0.05, **p < 0.01, ***p < 0.001, ****p < 0.0001).

To explore further the requirement for ATP in maintaining ROCK signalling, endothelial cells were exposed to the glycolysis inhibitor 2DG (20 mM) or the mitochondrial electron transport chain inhibitor rotenone (0.5 μM) 2 hours before CA4P; these were conditions previously shown to reduce intracellular ATP levels in HUVECs^33^. Individually, 2DG and rotenone significantly reduced basal and CA4P-induced levels of pMLC (Fig. 5a,b). pMLC was undetectable in control and CA4P-treated cells exposed to 2DG and rotenone simultaneously. Similar results to those obtained with rotenone were obtained with oligomycin, a different mitochondrial respiration inhibitor (not shown). AMPK was phosphorylated/activated in cells exposed to 2DG and/or rotenone. Levels of pHSP27 rose in the presence of either 2DG or rotenone, but in cells exposed to both inhibitors simultaneously, pHSP27 levels dropped down to basal (Fig. 5a). Similarly, pERK1/2 levels were reduced when energy levels were depleted. Τhe effects of ATP depletion on signalling were reversed when normal metabolism was restored by removing 2DG and rotenone and allowing the cells to recover in fresh culture media for 15 or 30 minutes (Fig. 5a,b). Levels of pMLC and pERK1/2 were restored while pAMPK declined back to basal (Fig. 5a). pHSP27 levels rose during the recovery period, possibly as a consequence of ischaemia/reperfusion stress. Similar to hypoxia, GTP-bound active RhoA was significantly reduced in cells exposed to 2DG and rotenone suggesting that ATP depletion interferes with the Rho-GTPase activation cycle (Fig. 5c,d).

**Figure 6:**
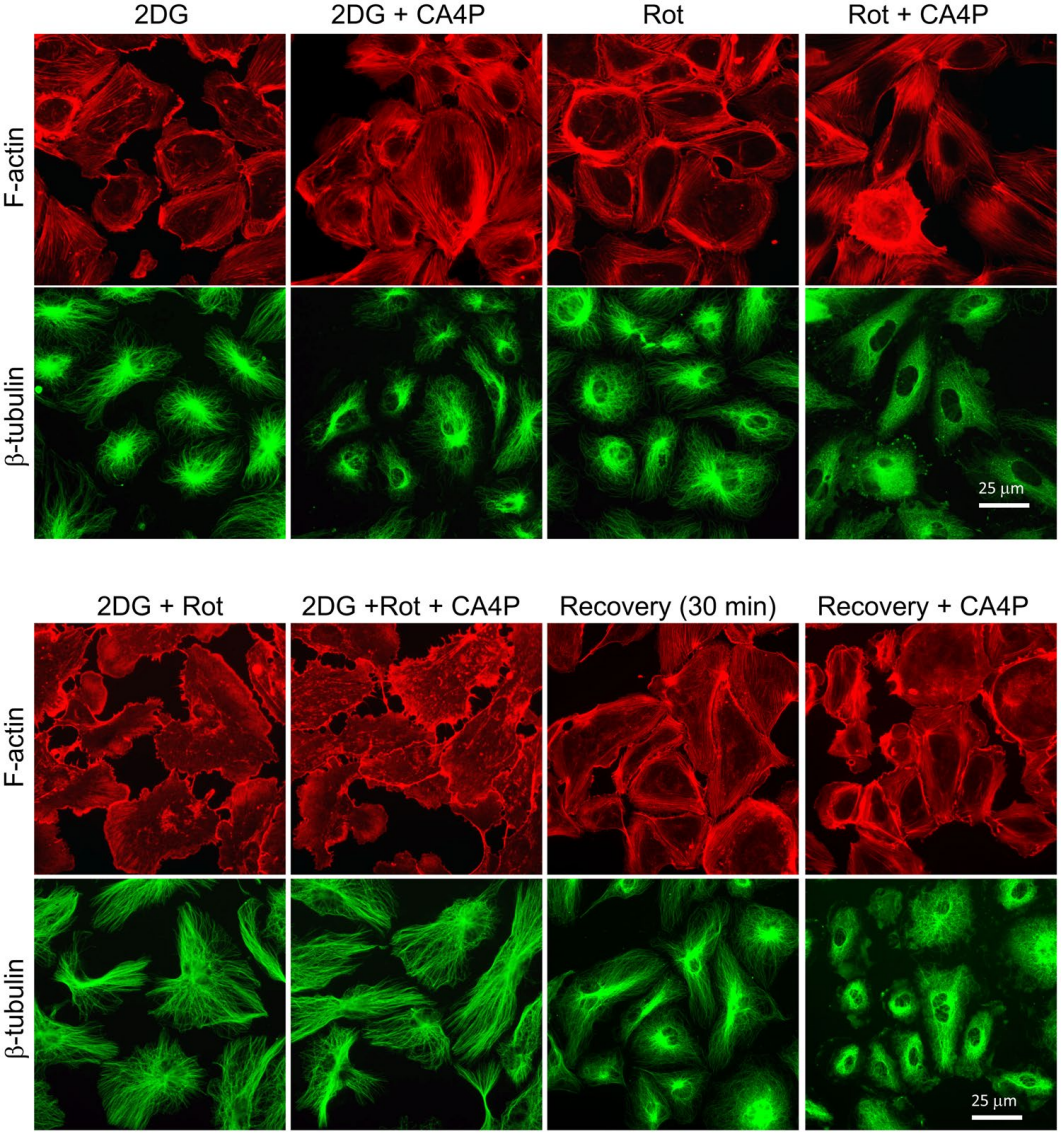
Inhibitors of glycolysis and oxidative phosphorylation inhibit F-actin filaments and stabilise microtubules against disruption by CA4P. Confluent endothelial cultures were incubated with vehicle control (Ctr) or 2DG (20 mM) or 0.5 μM rotenone (Rot) or 2DG plus rotenone for 1 hour. Cells were treated with CA4P (1 μM, 15 min) either immediately or after they recovered in fresh media for 30 min. Cells were fixed and double-stained with Texas Red phalloidin for F-actin (red) and β-tubulin (green).

Microtubules were resistant to CA4P disruption in cells treated with 2DG (compare images in Fig. 4c top panels to images in Fig. 6). When 2DG was combined with rotenone then microtubules became totally resistant to disruption while actin filaments were totally dissociated/absent (Fig. 6, lower panel). Actin filaments were restored rapidly following a period of recovery in fresh cell culture media (Fig. 6, lower panel). Similarly, microtubule sensitivity to CA4P was also re-established when ATP levels were restored. These results show that energy levels/ATP determine the endothelial cytoskeleton response to CA4P.

### The role of AMPK on endothelial response to CA4P

**Figure 7:**
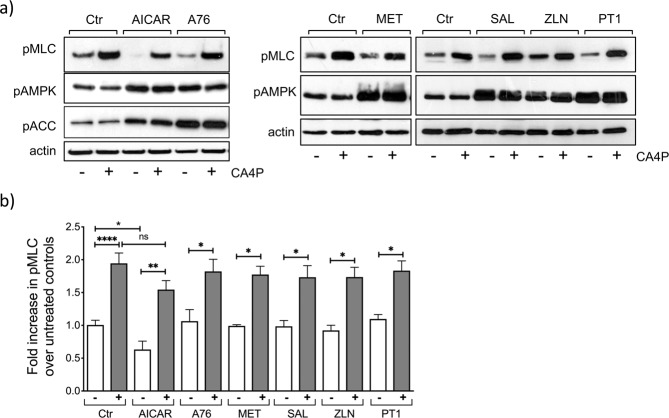
Pharmacological activators of AMPK do not influence pMLC induction by CA4P. (**a**) Confluent endothelial cultures were either left untreated as controls (Ctr) or incubated with AMPK activators: AICAR (1 mM), A-769222 (A76, 300 μM), salicylate (SAL, 5 mM), ZLN024 (ZLN, 40 μM), or PT1 (20 μM) for 1 hour, or metformin (MET, 2 mM) for 14 h. CA4P (1 μM) was then added for 15 min and cell extracts were analysed for expression of the indicated proteins. (**b**) pMLC was normalised to actin and expressed as fold increase over control untreated cells. Results are from 3–7 independent experiments. Data were analysed by one-way Anova followed by a Tukey post-test. Values are means ± SEM (*p < 0.05, **p < 0.01, ***p < 0.001, ****p < 0.0001).

AMPK exists as a heterotrimeric protein consisting of catalytic α and regulatory βγ subunits forming an AMPKα2β2γ3 complex that plays a central role in the regulation of cell metabolism. In addition, AMPK is known to influence signalling pathways associated with the cytoskeleton^23,34^ and in particular it was reported to regulate the activity of several members of the Rho-GTPase family^35^. In the next experiments, a number of pharmacological agents that activate AMPK through distinct mechanisms were used in order to establish whether AMPK itself played a direct role in altering RhoA/ROCK activation by CA4P. AMPK can be activated by agents that raise AMP/ATP levels or by compounds that directly bind it and act through conformational changes^36^. AICAR, a widely used small molecule AMPK activator, is converted to the AMP mimetic ZMP that binds to the γ subunit of AMPK and causes conformational changes leading to sustained phosphorylation of Thr-172 on the α subunit^37^. Several other small molecule activators of AMPK include the Abbott Laboratories compound A-769662^38^, salicylate^39^, PT1^40^ and ZLN024^41^ which are all reported to activate AMPK allosterically by binding directly to different sites on AMPK subunits. Metformin on the other hand activates AMPK indirectly by increasing the AMP/ATP ratio^42^. AICAR or A-769662, caused a substantial activation/phosphorylation of AMPKα and ACC in endothelial cells (Fig. 7a). AICAR significantly reduced basal levels of pMLC and there was also a trend for a reduction in pMLC levels in cells subsequently treated with CA4P, compared to cells treated in the absence of AICAR; however, the data did not reach statistical significance (Fig. 7a,b). A-769662, metformin, ZLN024, salicylate and PT1 did not alter either the basal or CA4P-induced pMLC levels despite being potent at activating AMPK (Fig. 7a,b).

**Figure 8:**
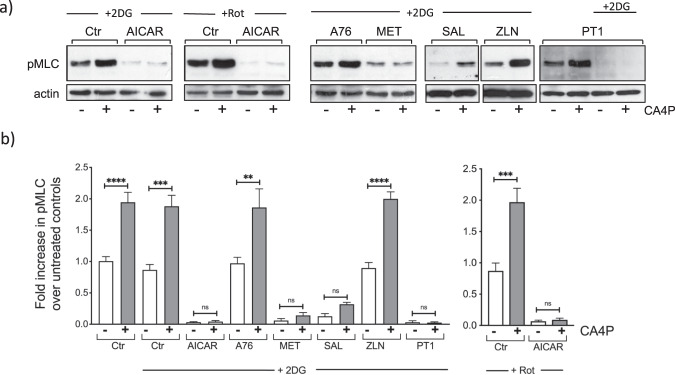
Only AMPK activators that generate AMP synergise with 2DG or rotenone to block CA4P-mediated ROCK signalling. (**a**) Cells were treated with AMPK activators: AICAR (1 mM), A-769222 (A76, 300 μM), salicylate (SAL, 5 mM), ZLN024 (ZLN, 40 μM) or PT1 (20 μM) for 1 hour, or metformin (MET, 2 mM) for 14 hours. 2DG (10 mM) or rotenone (Rot, 0.25 μM) were added to the cells at the same time as the AMPK activators except for in the case of metformin when 2DG was added during the last hour of incubation. CA4P (1 μM) was added for a further 15 min and cell extracts were analysed for pMLC. (**b**) pMLC was normalised to actin and expressed as fold increase over control cells without any drug treatments. Results are from 3–5 independent experiments. Data were analysed by one-way Anova followed by a Tukey post-test. Values are means ± SEM (*p < 0.05, **p < 0.01, ***p < 0.001, ****p < 0.0001).

In the next series of experiments, cells were exposed to each of the AMPK activators together with 10 mM 2DG. At this dose, 2DG in the absence of another AMPK activator, did not cause a substantial change in basal or CA4P-induced levels of pMLC. However, when combined with AICAR, 2DG synergised to substantially reduce pMLC (Fig. 8a,b). Because AICAR could potentially increase the uptake of 2DG and therefore further deplete the cells of ATP we also exposed cells to AICAR and rotenone. This combination also caused a complete reduction in pMLC (Fig. 8a,b) implying that an enhanced uptake of 2DG was an unlikely cause of the observed 2DG and AICAR synergism. Similar to AICAR, metformin, salicylate and PT1, also caused a substantial reduction in pMLC when combined with 2DG but this effect was not reproduced by A-769662 or ZLN024 (Fig. 8a,b). These data show that each pharmacological activator of AMPK influences Rho/ROCK signalling via unique mechanisms that are independent of activated AMPK itself.

## Discussion

In this study we found that hypoxia and energy depletion, diminished the ability of endothelial cells to respond to CA4P. Using the induction of phosphorylation of ROCK-effector MLC, as a measure of the activity of CA4P^8,9^, we showed that hypoxia, or conditions that depleted the cells of energy/ATP, reduced basal levels of pMLC and/or prevented the induction of MLC phosphorylation by CA4P. The decline in pMLC under hypoxia was paralleled by a reduction in RhoA-GTPase activity which is upstream of ROCK and MLC phosphorylation. Reduced remodelling of the actin cytoskeleton and reduced actinomyosin contractility, correlated with the extent of energy depletion. In hypoxia, the decline in pMLC was dependent on both the duration as well as the severity of oxygen depletion. Glucose deprivation caused a much faster reduction in pMLC compared to that caused by hypoxia, while hypoxia synergised with glucose deprivation and reduced pMLC levels further. Inhibitors of oxidative phosphorylation and glycolysis also reduced pMLC levels, and when combined in order to completely deplete cells of ATP, there was profound loss of pMLC and actin filaments were totally disrupted. Our data therefore provide strong evidence that the activity of the RhoA-ROCK-pMLC signalling axis and the regulation of the actin cytoskeleton, and hence the response of endothelial cells to CA4P, are directly dependent on intracellular ATP levels.

Many diverse signalling activities are regulated by hypoxia in endothelial and other cells. Specifically, hypoxia-mediated regulation of Rho family members, as seen by changes in their gene and protein expression levels, and/or through changes in their GTPase activities has been reported^43^. However, the activity and levels of expression of Rho proteins have been shown to either increase or decrease in hypoxia. Wojciak-Stothard *et al*.^44^ showed that in porcine endothelial cells RhoA-GTPase activity increased and peaked between 1–2 hours at 3% O_2_ and then declined back to basal levels by 4 hours. Similarly, in human pulmonary endothelial cells, active RhoA and RhoB were significantly elevated in 2% O_2_ and in parallel there was an increase in RhoA and RhoB gene and/or protein expression that peaked at early time points and reverted to basal levels by 4 hours^45^. Unlike Wojciak-Stohard *et al*.^44,45^, we found no significant changes in the extent of MLC phosphorylation and the ability of CA4P to phosphorylate it further at early time points (1–5 hours) following the initiation of hypoxia, suggesting that there were no substantial changes in the activity of RhoA or RhoB GTPases under our conditions. We used different endothelial cells and hypoxia conditions than Wojciac-Stothard *et al*. and this could potentially explain our different findings. On the other hand, Solodushko *et al*.^46^ showed that when rat and human pulmonary microvascular endothelial cells were cultured in 5% O_2_ compared to 21% O_2_ they formed tighter junctions and were less permeable due to a reduction in RhoA-GTPase activity. Recently, Leinhos *et al*.^47^ showed that Rho-GTPase activity was markedly downregulated in fibroblasts exposed to 1% oxygen. Rho-GTPases are regulated by GEFs (guanine exchange factors) that promote exchange of GDP for GTP and activate Rho proteins, and by GAPs (guanine activating factors) that facilitate the exchange of GTP for GDP that turn off the activity of Rho proteins. Reduced activity of RhoA was attributed to a selective induction in GAP ARHGAP29 expression under hypoxia^47^. It is possible that under our prolonged and severe hypoxic conditions (14 h at 0.1 or 1% O_2_), or under conditions of total energy depletion, a GAP protein(s) was induced, which may have contributed to the reduction in Rho-GTPase activity we reported here. This would also imply that GAP levels declined rapidly following re-oxygenation or recovery from ATP depletion in our system.

A drop in ATP levels in hypoxia can explain our results and there are several lines of evidence to support this. Low supplies of oxygen limit mitochondrial oxidative phosphorylation and reduce intracellular ATP levels as shown previously in HUVECs in 0.5 or 1.5% O_2_ (levels dropped by ≈27% and 20% respectively after 2 hours)^33^. Selective inhibition of RhoA-GTPase as a consequence of ATP depletion was previously demonstrated in epithelial cells^48,49^. The effects of ATP reduction were selective for RhoA, and not for other members of the Rho family, namely Rac1 and Cd42, and it was proposed that the preferential selectivity for RhoA was due to effects on specific GEFs and GAPs. Additionally, in astrocytes, ATP activated RhoA rapidly, providing direct evidence for the involvement or ATP in the regulation of Rho-GTPases^50^. Endothelial cells can use glycolysis to generate ATP and limited supplies of nutrients and glucose often accompany hypoxia *in vivo*. We hypothesised that if the reduction in RhoA/ROCK activity in hypoxia was due to lower levels of ATP, then glucose deprivation would mimic the effects of hypoxia. Indeed, pMLC downregulation was substantial in glucose-deficient media and pMLC was further reduced by exposure to hypoxia suggesting that the two synergised to reduce ATP levels further. Indeed, Quintero *et al*.^33^ showed that if 2DG and hypoxia were combined, then by 2 hours, ATP levels were substantially reduced (they dropped by ≈63% in 1.5% O_2_ and by ≈75% in 0.5% O_2_), and therefore our results could be explained by a further reduction in ATP levels when glucose deprivation and hypoxia were combined. Levels of pMLC also dropped by inhibiting either glycolysis or oxidative phosphorylation under identical conditions to those used by Quintero *et al*.^33^. A more drastic and profound loss of pMLC, however, occurred after exposing the cells simultaneously to 2DG and rotenone, these being conditions that led to almost total ATP loss in HUVEC (ATP levels dropped by ≈95% in 2 hours)^33^. Conversely, when ATP levels were restored by allowing the cells to recover in fresh full media, then a rapid recovery of pMLC signalling and cytoskeletal structures was seen (Figs. 5 and 6). Our data therefore point to a direct link between intracellular levels of ATP, pMLC and consequently actin regulation.

The energy sensor kinase AMPK itself has been implicated in the regulation of the cytoskeleton^23,24,35^. Xing *et al*.^35^ showed that lipopolysaccharide-induced RhoA/ROCK activation in lung endothelial cells was inhibited by the AMPK activator AICAR. We sought to establish whether activated AMPK was the common link between lowered ATP and loss of ROCK signalling. We hypothesised that if AMPK was directly responsible for lowering ROCK activity, then pharmacological activators of the kinase would mimic the effects of low ATP. However, all the pharmacological AMPK activators we tested caused robust activation of AMPK, but only AICAR caused a significant reduction in basal levels of pMLC. Furthermore, AICAR and the other AMPK activators that were tested failed to alter the induction of pMLC by CA4P (see Fig. 7). These results suggest that AMPK activation *per se* is insufficient to downregulate pMLC and regulate the cytoskeleton. AICAR generates the AMP-mimetic ZMP, which accumulates at high concentrations, and binds competitively at the AMP/ATP/ADP regulatory site on AMPKγ to activate the kinase^37^. Metformin inhibits mitochondrial complex I and alters the ratio of AMP/ATP, and this in turn activates AMPKγ through binding of AMP to the AMP/ATP/ADP regulatory binding site^42^. However, A-769662 does not generate AMP, binds AMPKγ at a different site and activates the enzyme allosterically^38^. Similarly, ZLN024, PT1 and salicylate are reported to activate AMPK allosterically. We found that while metformin and AICAR synergised with 2DG to substantially reduce pMLC levels in endothelial cells, A-79662 did not. Salicylate and PT1 also synergised with 2DG to reduce pMLC while ZLN024 behaved similar to A-79662. Being direct allosteric activators of AMPK, salicylate and PT1 were not initially considered to influence AMP and ADP. Nevertheless, these agents were subsequently shown to also alter mitochondrial respiration and increase the AMP/ATP ratio in cells^39,51^. It appears therefore, that only the AMPK activators that can also generate AMP, or the AMP mimetic ZMP in the case of AICAR, are capable of synergising with the metabolism inhibitors and alter RhoA/ROCK signalling. It is therefore likely that sensing of lowered ATP levels (or an increase in the ratio of AMP/ATP) beyond a certain threshold translates to a reduction in RhoA/ROCK activity, through an as yet unidentified mechanism(s) that is nevertheless likely to be independent of AMPK.

ROCK-dependent signalling by CA4P induces actin remodelling, and contractility-driven membrane ‘blebbing’^8^. Under hypoxic conditions, highly contractile ‘blebbing’ cells did not form after CA4P. The reduction in contractility was also associated with a profound reduction in VE-cadherin junctional disruption and reduced permeability to dextran. Similarly, if cells were deprived of glucose, then the development of highly contractile ‘blebbing’ cells was abolished. However, stress fibers were present under hypoxic and glucose depleted conditions, even when pMLC levels were extremely low and cells would not be expected to form stress fibers. Hypoxia affects many signalling pathways and can alter stress response proteins including members of the mitogen activated protein family^32^. Indeed, hypoxia altered the phosphorylation of ERK1/2 and HSP27 in endothelial cells. Induction of pERK1/2 occurred at 1% O_2_ but levels dropped significantly at 0.1% O_2,_ while HSP27 phosphorylation was more pronounced at 0.1% O_2_. When phosphorylated, ERK1/2 can protect HUVECs from ROCK-induced ‘blebbing’ as we showed before^8^, while activated HSP27 can promote actin filaments and stress fibres to form in the absence of activated ROCK signalling. The phosphorylation status of HSP27 correlated with both absence of ‘blebbing’ and the presence of stress fibres seen after hypoxia (Fig. 3b) when levels of active pMLC were low. Cells completely lost their stress fibres and pHSP27 if both glycolysis and oxidative phosphorylation were blocked, these being conditions of extreme ATP depletion (see Fig. 6). Previous reports showed a similar loss of actin filaments/stress fibers in epithelial cells after ATP depletion^48^.

Re-oxygenation, following hypoxia has been reported to result in changes in Rho protein-dependent signalling. Wojciac-Stothard *et al*.^44^ showed that re-oxygenation increased Rac1-GTPase activity through the production of ROS and reduced RhoA-GTPase activity in porcine endothelial cells, while in contrast, others showed that re-oxygenation increased levels of RhoA activity in porcine endothelial cells with a concomitant reduction in Rac1 activity^52^. We found that re-oxygenation restored the ability of CA4P to activate ROCK-mediated signalling and the ability of CA4P to activate it further. Both Rac and Rho-GTPases can be activated by ROS while Rac1 itself can generate ROS through the activity of NADPH oxidase^53^. Rac1 and Rho antagonise each other^54^ so that a reciprocal balance between their GTPase activities exists. The precise consequences of ROS generation on the activities of individual members of the Rho-GTPase family are therefore likely to depend on the specific cellular system, local stimuli or other factors. The rapid recovery of ROCK signalling by reoxygenation in our study can be explained by both an increase in ATP levels through restored mitochondrial activity as well as by ROS-dependent activation of RhoA signalling.

We also saw substantial changes in microtubule stability under conditions of energy depletion. Microtubules became totally resistant to CA4P disruption under conditions of severe ATP depletion (see Fig. 6). Similar effects of ATP depletion were previously reported for fibroblasts^55^. Hypoxia is also thought to regulate microtubule stability and render microtubules resistant to disruption^56^. Although we did not observe any substantial differences in the susceptibility of endothelial microtubules to CA4P when cells were maintained in hypoxia *versus* normal oxygen conditions (data not shown), we nevertheless observed more intact microtubules after CA4P in glucose-deprived cultures concurrently exposed to hypoxia compared to cultures maintained in normal oxygen and glucose (see Fig. 4b). The mechanisms through which ATP depletion stabilises microtubules are unclear but may relate to regulation of tubulin by post-translational modifications such as tyrosination and acetylation or enhanced expression of proteins including ERG-1^55–57^.

The microtubule cytoskeleton of endothelial cells is the initial target of CA4P and other similar VDAs. Induction of contractility and disruption of cell-cell junctions and permeability rise that occur soon after the disruption of microtubules are considered important *in vivo* as part of the mechanism of vascular shutdown by CA4P and similar VDAs^1^. The changes we describe in this study under hypoxia and in conditions of energy depletion may translate into resistance to CA4P *in vivo*. This is provided oxygen and ATP levels within the tumour vessels drop below a certain threshold required for efficient microtubule disruption and activation of ROCK signalling by CA4P. Intravascular hypoxia occurs in tumours through deficiencies in oxygen transport and vessels can be hypoxic even if they are flowing^58–60^. Longitudinal gradients of oxygen develop and intravascular pO_2_ drops as red blood cells move further from a supplying arteriole and this can result in regions where the pO_2_ becomes very low^59^. On the other hand, hypoxia may fluctuate, so cycles of hypoxia and reoxygenation occur with frequencies of a few cycles per hour to days^60^. Depending on the kinetics of cycling hypoxia, intravascular regions may remain poorly oxygenated for substantial periods of time^61^. Although our results do not explain why tumours that are generally considered hypoxic are more sensitive to VDAs than normal tissues, the consequences of frequent hypoxia-reoxygenation cycles may sensitise the vasculature to VDA-induced damage. Reactive oxygen species activate ROCK dependent mechanisms in the heart and other organs and are responsible for ischaemia-reperfusion injury^62^. Cycling hypoxia could therefore have a sensitising effect on the tumour vasculature to CA4P.

There are many factors within the tumour microenvironment that could potentially contribute to tumour sensitivity to VDAs. Interactions between stroma and tumour cells within the tumour microenvironment as well as systemic effects could well impact on the outcome of this type of therapy. We found that immature blood vessels lacking adequate pericyte coverage are more sensitive to VDA disruption^11^. Research from our team also showed that the vascular damaging effects of CA4P could be significantly enhanced by inhibiting constitutive isoforms of nitric oxide synthase^63^. Further studies are required to establish whether response to VDAs is modified by the continuously changing levels of oxygenation in tumours. If so, approaches to modify tumour oxygenation, similar to those undertaken for improving radiotherapy outcomes^64^, may also be explored as potential modifiers of VDA response.

## Methods

### Reagents

Rotenone, AICAR (5-aminoimidazole-4-carboxamide riboside), A-769662, metformin, ZLN024 and PT1 were purchased from Tocris and 2-deoxy-D-glucose (2DG) was purchased from Abcam. Oligomycin and antibodies to MLC, pMLC, pHSP27, pAMPKα, pACC were purchased from Cell Signaling Technology. Salicylate was purchased from Sigma. The anti-VE-cadherin and ROKα antibodies were from BD-Biosciences and the anti-RhoA antibody was from Merck Millipore. Glucose-free DMEM and dialysed serum were purchased from Gibco Life Technologies (Paisley, United Kingdom).

### CA4P synthesis

**Figure 9:**
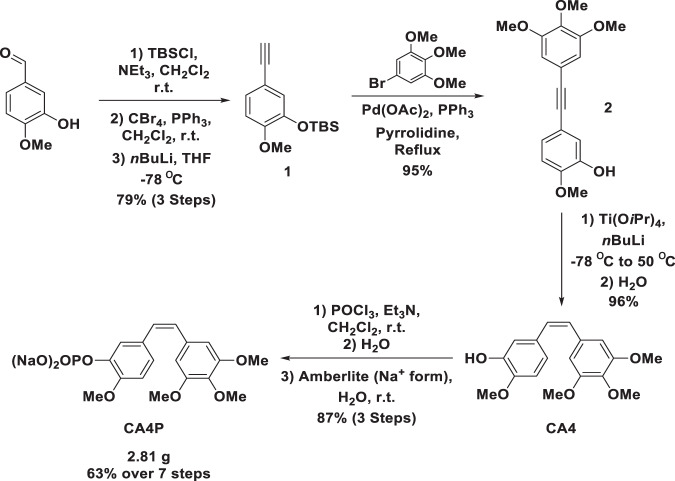
Scheme illustrating steps in CA4P synthesis.

CA4P was synthesized using a modified version of Lara-Ochoa’s protocol^65^. Alkyne **1** was prepared via Corey-Fuchs methodology. The reaction proceeded smoothly to yield **1**, in 79% yield, over three steps from isovanillin. The reaction was scalable to 130 mmol without affecting yield. Lara-Ochoa and co-worker had previously reported the copper-free Sonogashira coupling of the non-protected form of alkyne **1**, with 1-iodo-3,4,5-trimethoxybenzene in excellent yield^65^. Application of the reported conditions to **1** with 1-bromo-3,4,5-trimethoxybenzene resulted in the isolation of diarylalkyne **2** in a yield of 95%. Conveniently, the silyl ether was cleaved under the reaction conditions to yield the free phenol. Conditions from the same authors facilitated the selective reduction of alkyne **2** to CA4 with titanium(IV) tetraisopropoxide and *n*-butyl lithium (nBuLi). However, subjection of **2** to these conditions resulted in incomplete conversion and an inseparable mixture of starting material and product in our hands. It was found that, deprotonation of the phenol with an extra equivalent of *n*BuLi, before addition of the titanium reagent and further *n*BuLi, resulted in complete conversion of the starting material and isolation of CA4 in 96% yield, >98:2 Z:E. The crude product was of high enough purity to be taken forward to the next step without further purification. CA4 was successfully phosphorylated with phosphorus oxychloride in dichloromethane. It was found that a “one pot” protocol, in which the ion exchange resin was added to the crude isolated phosphate monoester in water, resulted in product isolation in an 87% yield after recrystallization from acetone. The purity of the product by HPLC was determined to be >99%. Therefore, CA4P was successfully synthesised, in gram quantities, in a 63% yield over 7 steps (Fig. 9).

### Cell culture

Human umbilical vein endothelial cells from pooled donors (Promocell, Germany) were grown on gelatin-coated culture dishes in ready-use Endothelial Cell Growth Medium (Promocell) supplemented with an additional 8% low endotoxin heat inactivated fetal calf serum (Invitrogen). Confluent monolayers were obtained by plating cells at a density of 2 × 10^4^ cells/cm^2^ and allowing them to grow for up to 7 days. Cultures were used 3–4 days after reaching confluence and were used for experiments for up to 5 passages. Assessment of cell viability was performed by the trypan blue exclusion method using the Biorad TC20^TM^ automated cell counter.

### Hypoxic conditions

Cells were incubated under hypoxic conditions in a Don Whitley VA500 anaerobic workstation (Don Whitley, Shipley, West Yorkshire) modified to accommodate individual sealed humidified chambers. Each chamber was fitted with an independent gas supply of 21%, 5%, 1% or 0.1% O_2_, with 5% CO_2_ and balance of nitrogen. All gases were certified to medical standards. The main workstation chamber was maintained as an anaerobic atmosphere so that when cultures were removed from their individual chambers for the addition of CA4P they were not exposed to oxygen. The O_2_ concentration in the chambers was determined using a hand-held O_2_ analyzer and O_2_ in cell culture media was checked using a Jenway 970 DO_2_ oxygen probe.

### Glucose and ATP deprivation and recovery experiments

To deplete cultures of glucose, monolayers were rinsed twice in glucose-free DMEM and then incubated in glucose-free DMEM supplemented with 5% dialysed FCS. In parallel, control cultures were treated in the same way except that glucose containing DMEM was used. ATP depletion was achieved by the addition of 2DG and/or rotenone to the cells without prior media change. To assess recovery from ATP depletion three consecutive media washes were performed and cells were incubated for a further 15–30 min in fresh media in a 37 °C incubator.

### Rho-GTPase activity assay

The Active Rho Pull Down Detection Kit (Thermo Scientific) was used to assess the activation status of RhoA-GTPase according to the manufacturer’s instructions. Briefly, proteins were extracted in lysis buffer supplied by the kit (ice cold and supplemented with protease inhibitors). Equal amounts (800 μg) of clarified protein were incubated with GST-Rhotekin-RBD and glutathione resin for 1 h at 4 °C. Active Rotekin-RBD-bound RhoA was eluted from the beads and analysed by western blotting using an anti-RhoA antibody supplied in the kit.

### Western blotting analysis

Cell lysates were prepared by lysing the cells in 63 mM Tris-HLC pH 6.8 and 2% SDS buffer. Extracts were further prepared in reducing Laemmli sample buffer (Bio-Rad) heated to 70 °C for ten minutes, resolved by electrophoresis on polyacrylamide gels and transferred to nitrocellulose or PVDF membranes. Membranes were probed with primary antibodies overnight at 4 °C followed by secondary antibodies coupled to HRP (Dako). Immunoreactive bands were visualized using enhanced chemiluminescence with EZ-ECL (GeneFlow) and developed on blue autoradiography film. Films were scanned and imported into Adobe Photoshop, converted into Grayscale and levels were adjusted using the ‘Auto’ function. The relevant lanes were cropped and assembled into multi-panel Figures. All full-length uncropped blots are shown as supplementary data.

### Immunofluorescence analysis of the cytoskeleton

HUVECs were cultured on fibronectin-coated Permanox Lab-Tek chamber slides (Life Technologies, Paisley, United Kingdom) and stained for actin filaments, microtubules and VE-cadherin junctions as we described before^8^. Immunofluorescence localization of β-tubulin and VE cadherin were performed simultaneously with F-actin staining using Texas Red conjugated phalloidin (Invitrogen). Cells were viewed with an Olympus BX microscope and images were captured using Cell^F software and processed using Adobe Photoshop.

### Measurement of endothelial permeability

Diffusion of FITC-coupled 40 kDa dextran through endothelial monolayers was determined in HUVECs grown to confluence on fibronectin-coated culture inserts (3-μm pore size; 0.3 cm^2^ surface area) set into 24-well companion plates (BD Biosciences) as we described before^8^. Confluent cells on filters were placed inside the chambers of the anaerobic workstation for a further 14 h before they were treated with CA4P for 20 min and then FITC-coupled dextran was added and media samples were collected from the lower compartment 30 min later. Fluorescence was quantified using a Galaxy FLUOStar microplate reader and results are expressed as arbitrary fluorescence normalized against values obtained from parallel control cells maintained in 21% O_2_.

### Statistical analysis

Data were analyzed by either unpaired t-test or one-way ANOVA followed by Tukey’s analysis for multiple comparisons using Prism 5 software (Graphpad software). A value of P < 0.05 was considered significant.

## Supplementary information

Supplementary Figures.
