# Supplementary material for: The influence of hypoxia and energy depletion on the response of endothelial cells to the vascular disrupting agent combretastatin A-4-phosphate
Source: Sci Rep. 2020 Jun 18;10:9926. doi: 10.1038/s41598-020-66568-8 (PMC7303175; doi:10.1038/s41598-020-66568-8)
Supplement: Supplementary file 1 — Supplementary Figures. [file 41598_2020_66568_MOESM1_ESM.pdf]

# The influence of hypoxia and energy depletion on the response of endothelial cells to the vascular disrupting agent combretastatin A-4-phosphate

Toby Holmes, Andrew W. Brown^, Marie Suggitt, Lucy A. Shaw, Lucy Simpson, Joseph P. A. Harrity^, Gillian M. Tozer* and Chryso Kanthou*.

Figure 1a

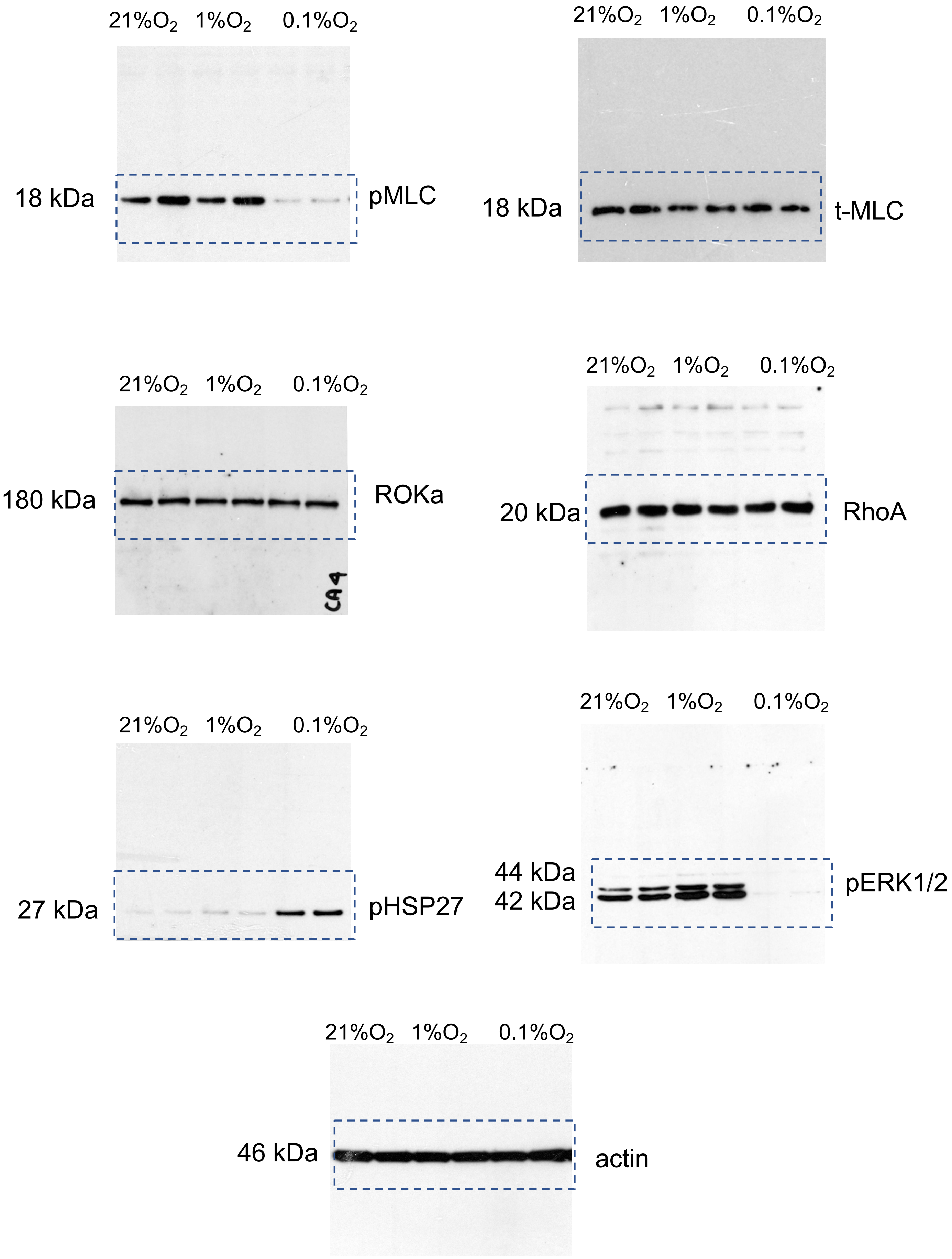

21%O2 1%O2 0.1%O2
18 kDa
pMLC
21%O2 1%O2 0.1%O2
18 kDa
t-MLC
21%O2 1%O2 0.1%O2
180 kDa
ROKa
21%O2 1%O2 0.1%O2
20 kDa
RhoA
21%O2 1%O2 0.1%O2
27 kDa
pHSP27
21%O2 1%O2 0.1%O2
44 kDa
42 kDa
pERK1/2
21%O2 1%O2 0.1%O2
46 kDa
actin

Figure 1c

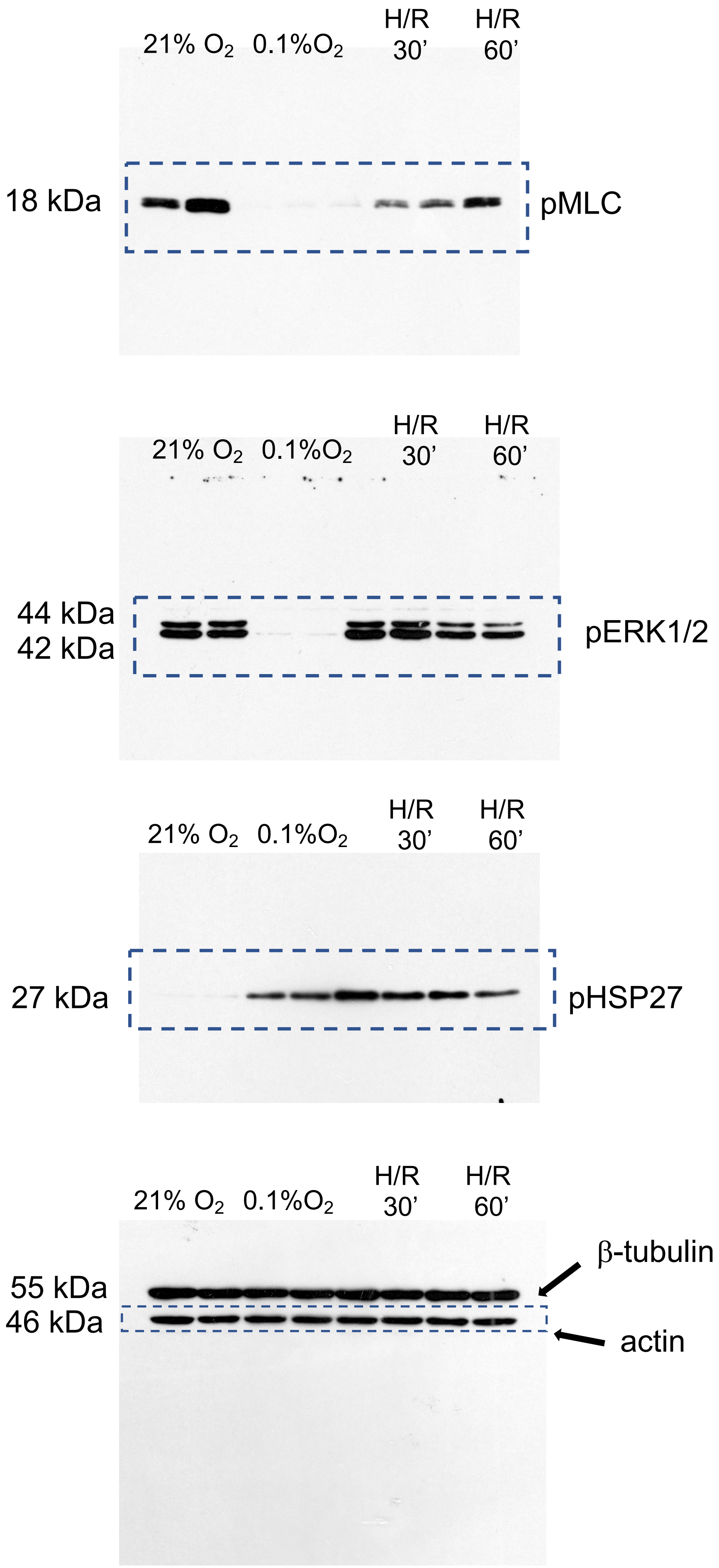

21% $O_2$
0.1%$O_2$
H/R 30'
H/R 60'
18 kDa
pMLC
21% $O_2$
0.1%$O_2$
H/R 30'
H/R 60'
44 kDa
42 kDa
pERK1/2
21% $O_2$
0.1%$O_2$
H/R 30'
H/R 60'
27 kDa
pHSP27
21% $O_2$
0.1%$O_2$
H/R 30'
H/R 60'
55 kDa
46 kDa
β-tubulin
actin

Figure 1d

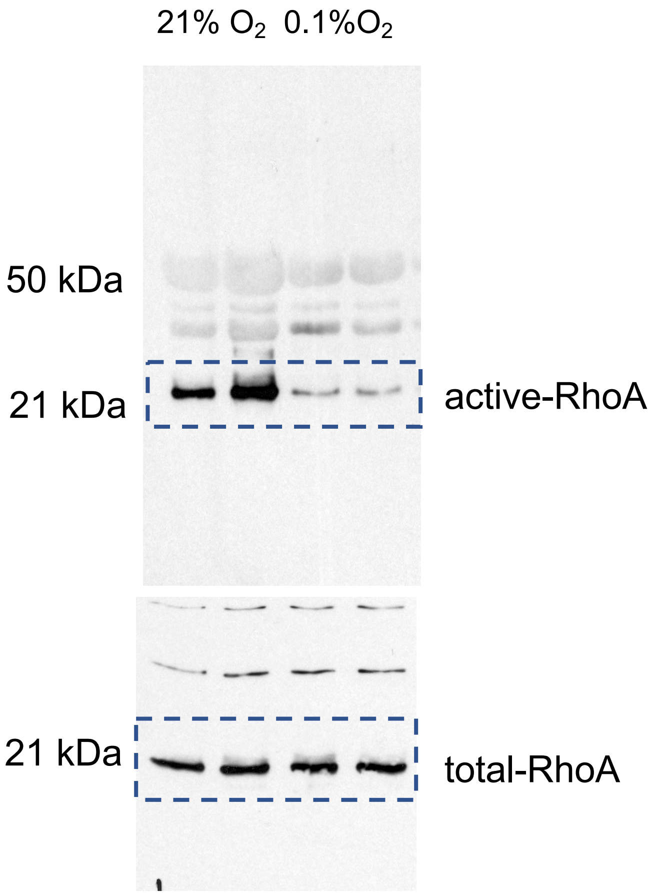

Figure 4a

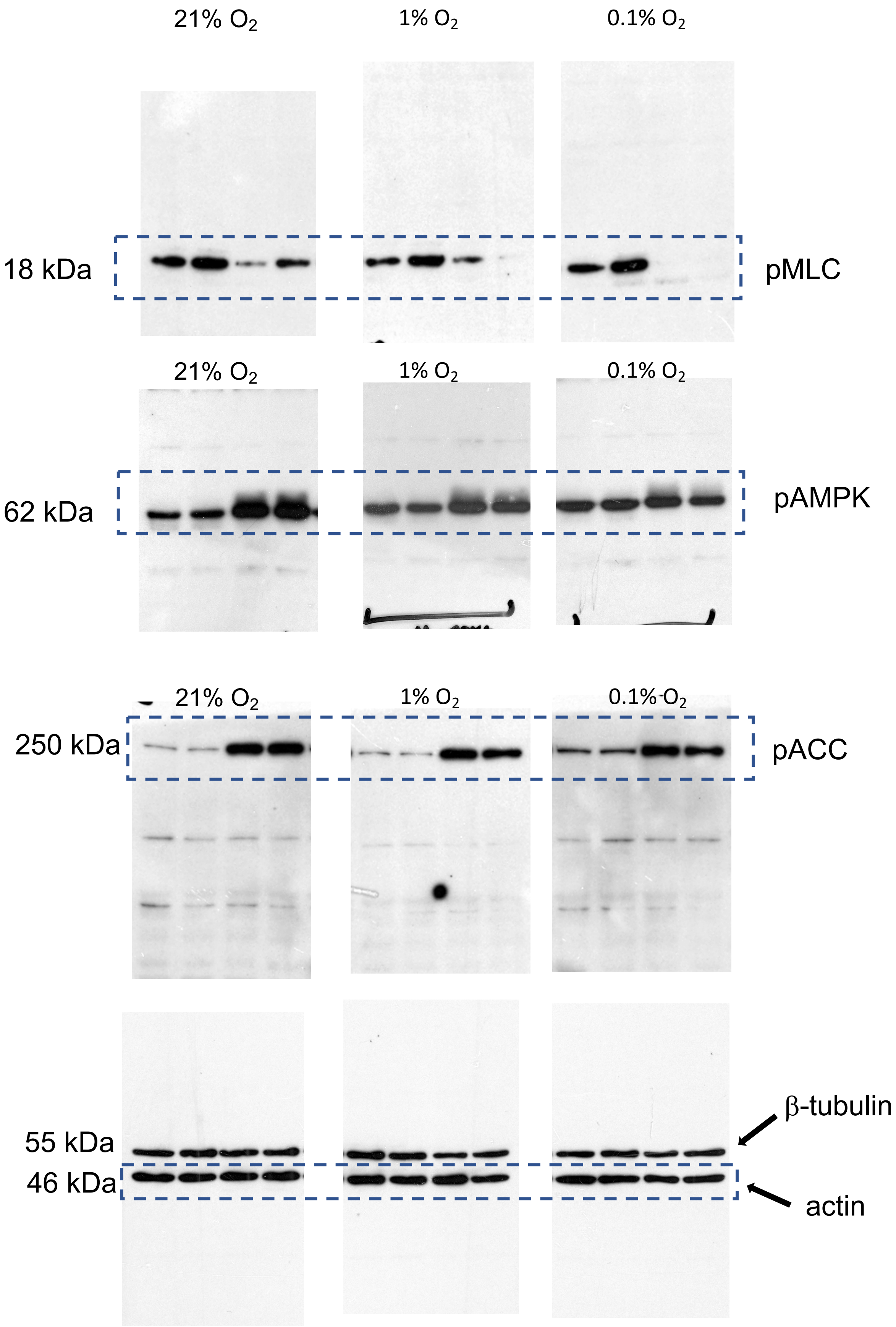

21% O$_2$
1% O$_2$
0.1% O$_2$
18 kDa
pMLC
21% O$_2$
1% O$_2$
0.1% O$_2$
62 kDa
pAMPK
21% O$_2$
1% O$_2$
0.1% O$_2$
250 kDa
pACC
55 kDa
46 kDa
β-tubulin
actin

Figure 5a left panel

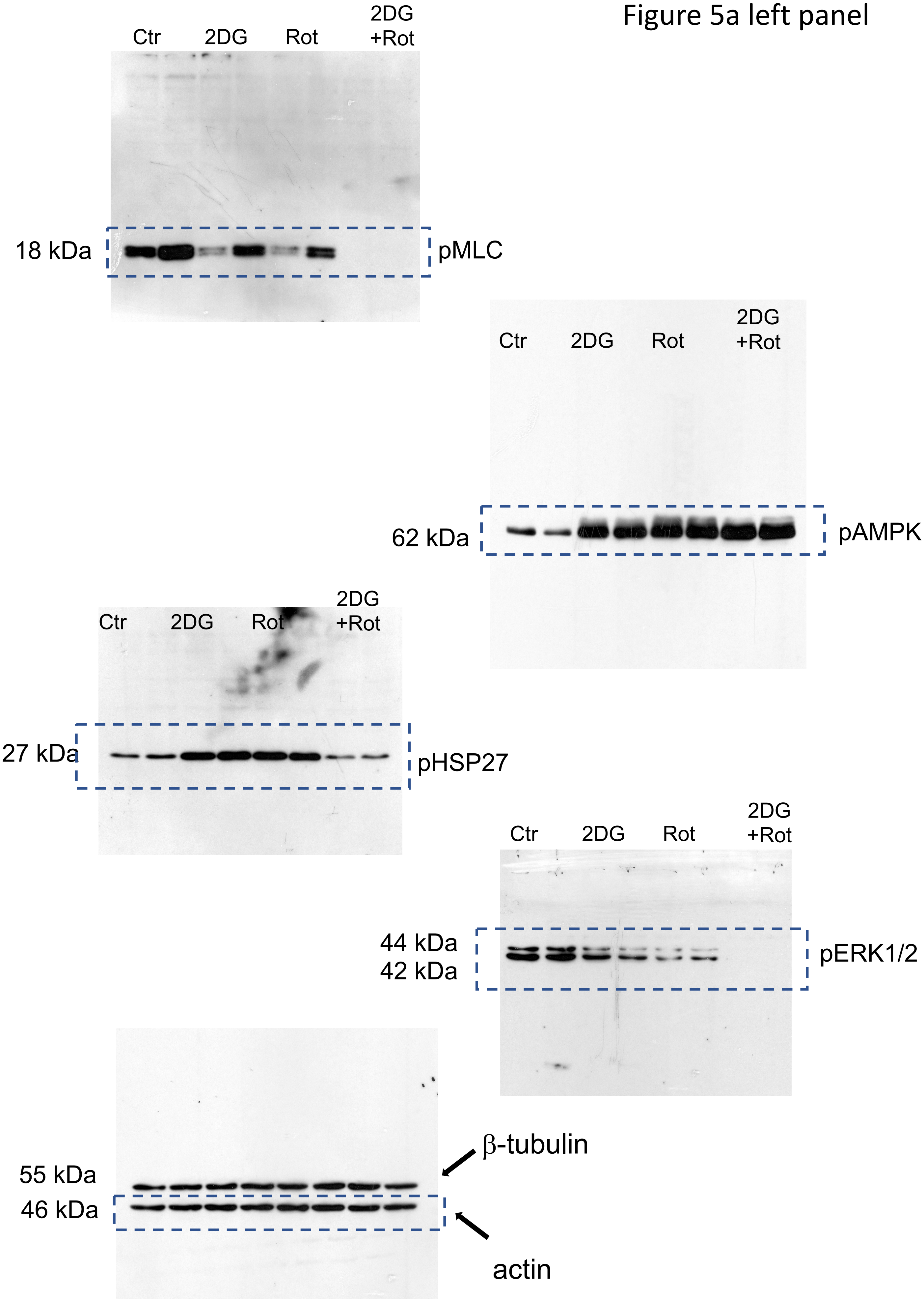

Figure 5a right panel

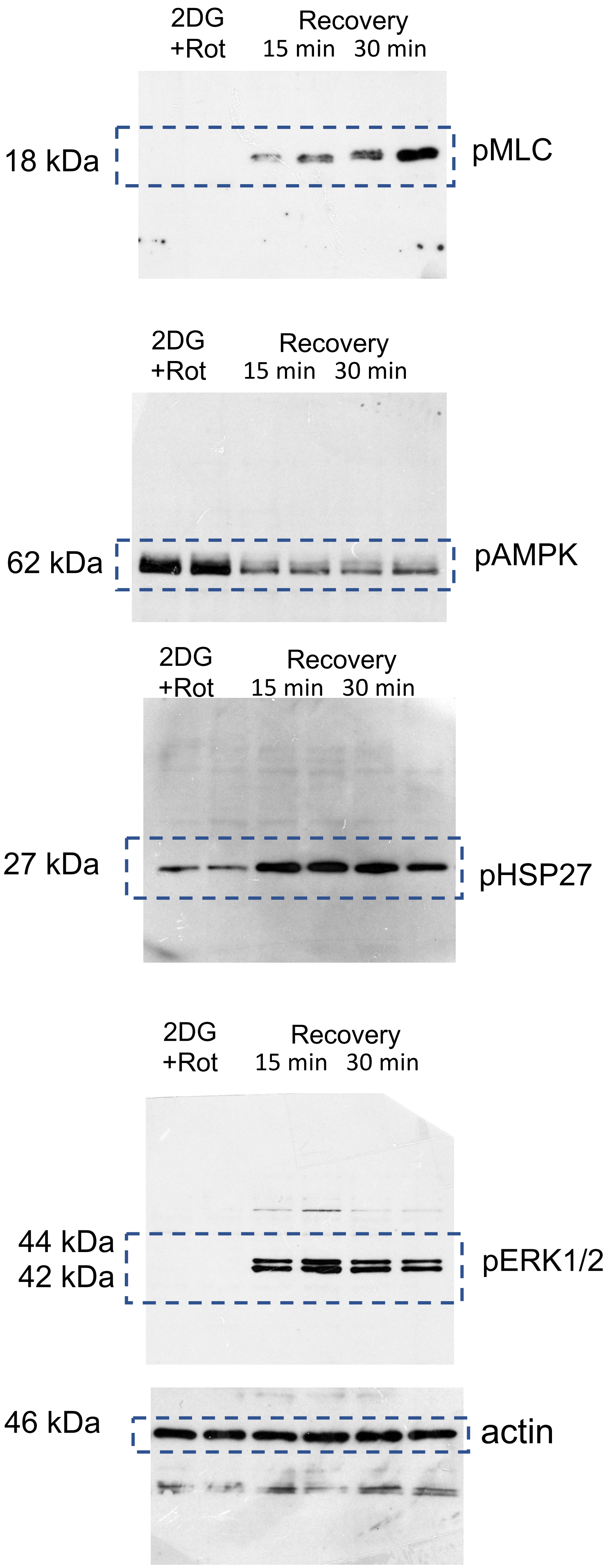

# Figure 5c

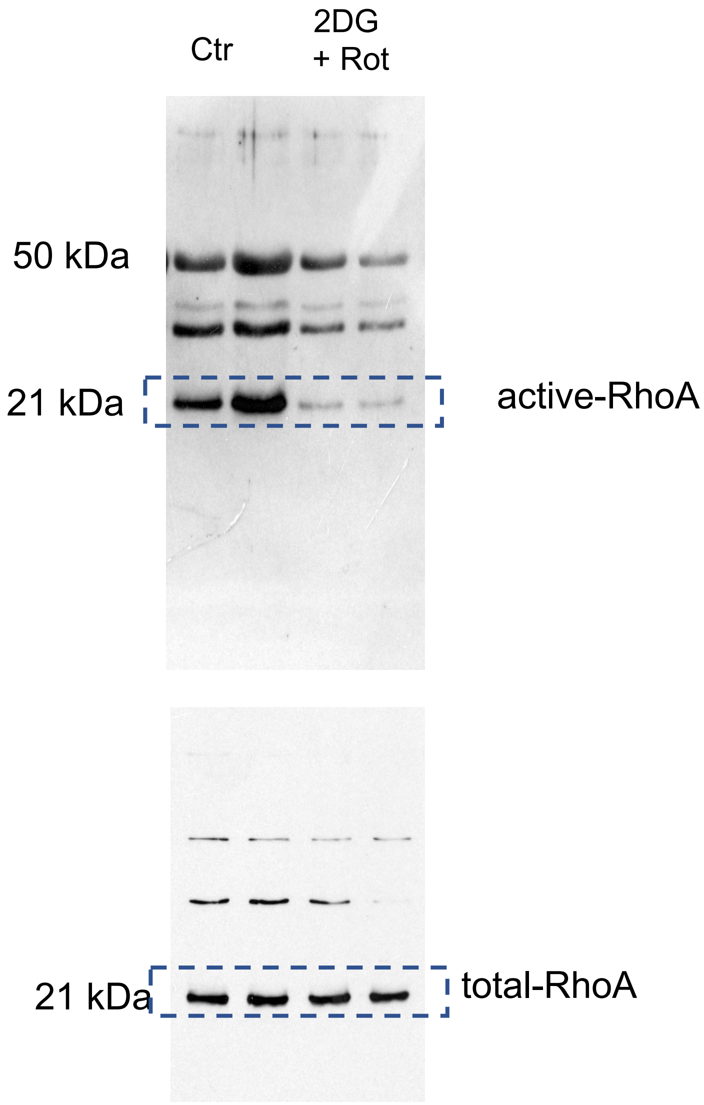

Figure 7a – left panel

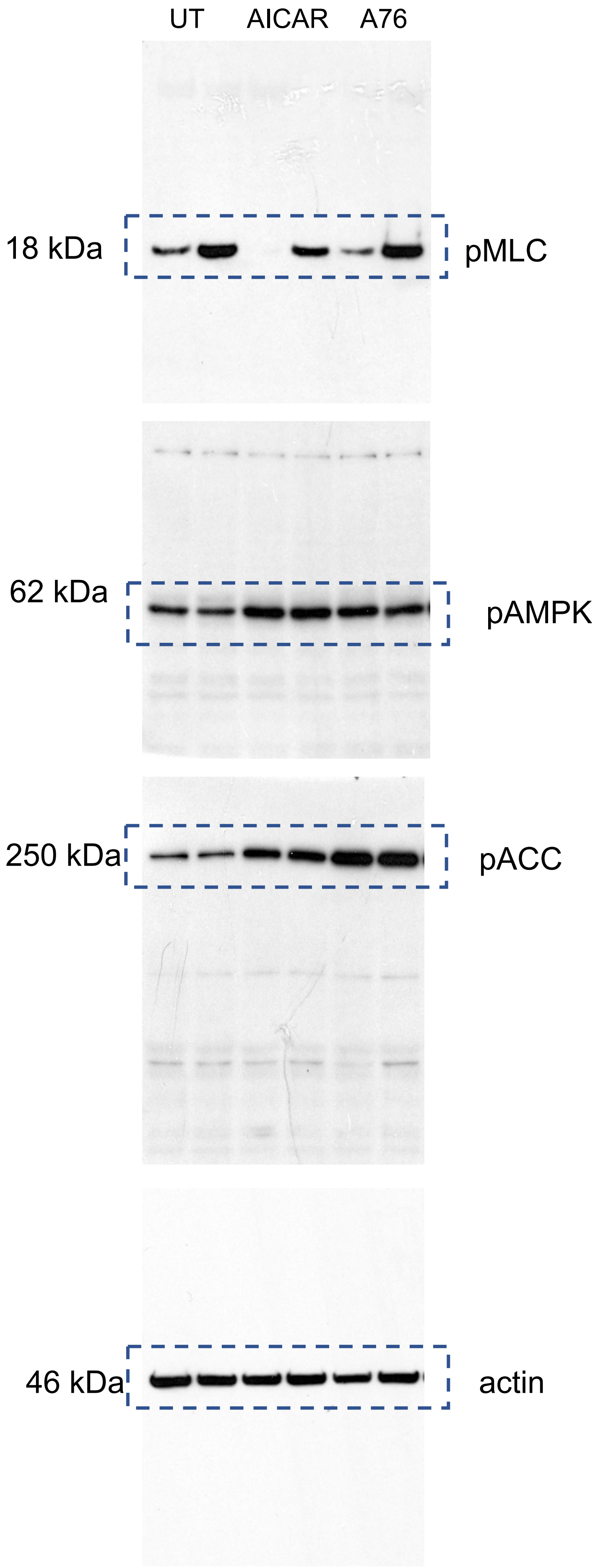

# Figure 7a – right panel

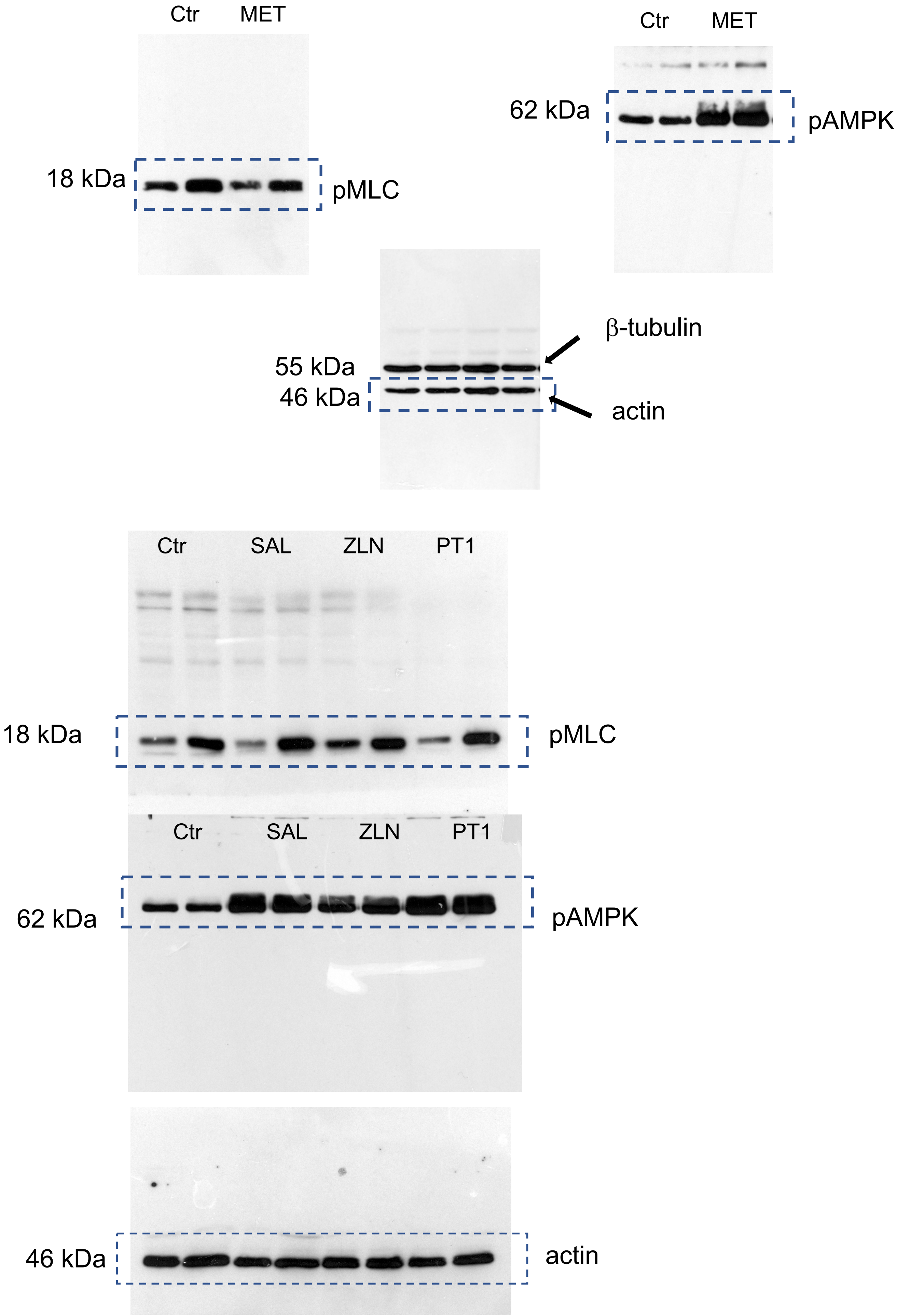

Figure 8a

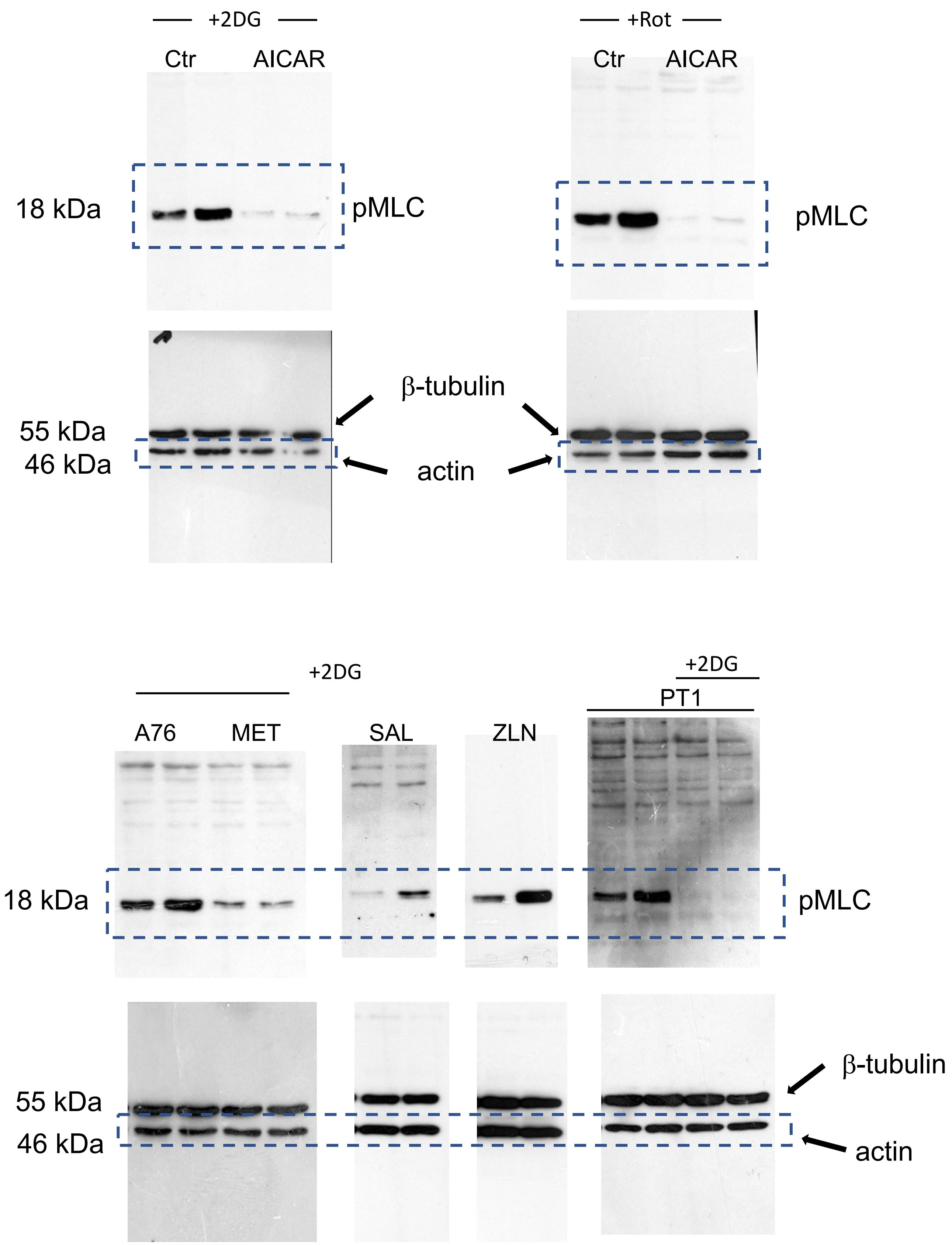

+2DG
Ctr
AICAR
+Rot
Ctr
AICAR
18 kDa
pMLC
pMLC
β-tubulin
55 kDa
46 kDa
actin
+2DG
+2DG
PT1
A76
MET
SAL
ZLN
18 kDa
pMLC
β-tubulin
55 kDa
46 kDa
actin
